# Two Highly Similar Poplar Paleo-subgenomes Suggest an Autotetraploid Ancestor of Salicaceae Plants

**DOI:** 10.3389/fpls.2017.00571

**Published:** 2017-04-12

**Authors:** Yinzhe Liu, Jinpeng Wang, Weina Ge, Zhenyi Wang, Yuxian Li, Nanshan Yang, Sangrong Sun, Liwei Zhang, Xiyin Wang

**Affiliations:** ^1^School of Life Science, North China University of Science and TechnologyTangshan, China; ^2^Center for Genomics and Computational Biology, North China University of Science and TechnologyTangshan, China

**Keywords:** poplar, grape, fractionation, genome alignment, gene collinearity, genomic homology, polyploid

## Abstract

As a model plant to study perennial trees in the Salicaceae family, the poplar (*Populus trichocarpa*) genome was sequenced, revealing recurrent paleo-polyploidizations during its evolution. A comparative and hierarchical alignment of its genome to a well-selected reference genome would help us better understand poplar’s genome structure and gene family evolution. Here, by adopting the relatively simpler grape (*Vitis vinifera*) genome as reference, and by inferring both intra- and inter-genomic gene collinearity, we produced a united alignment of these two genomes and hierarchically distinguished the layers of paralogous and orthologous genes, as related to recursive polyploidizations and speciation. We uncovered homologous blocks in the grape and poplar genomes and also between them. Moreover, we characterized the genes missing and found that poplar had two considerably similar subgenomes (≤0.05 difference in gene deletion) produced by the Salicaceae-common tetraploidization, suggesting its autotetraploid nature. Taken together, this work provides a timely and valuable dataset of orthologous and paralogous genes for further study of the genome structure and functional evolution of poplar and other Salicaceae plants.

## Introduction

Poplar (*Populus trichocarpa*), a Salicaceae plant, is important for providing raw fuel material for the manufacturing industry and it also plays an important ecological role in protecting the natural environment (Wang, 2007). Poplar was the first perennial woody plant to have had its whole genome sequence deciphered (Tuskan et al., 2006), and recently another Salicaceae plant, the willow, *Salix vitellina*, was likewise sequenced (Dai et al., 2014).

Polyploidy is an important genetic phenomenon of land plants, and it possibly contributed to their evolutionary origins and diversifications (Paterson et al., 2004; Soltis et al., 2008; Jiao et al., 2011). It is considered one of the main factors in the formation of the angiosperms, a large flowering plant group wherein recurring polyploidizations and extensive genome rearrangements rewire the combination of genes (Tang et al., 2008a). Dicotyledonous plants are likely to have originated from a common paleo-hexaploidy, as first revealed by analyzing the genome of Arabidopsis (*Arabidopsis thaliana*) (Bowers et al., 2003) and grape (*Vitis vinifera*) (Jaillon et al., 2007), and further confirmed by subsequent sequencing of pear (*Pyrus bretschneideri*), apple (*Malus × domestica*), strawberry (*Fragaria vesca*), peach (*Prunus persica*), and plum (*Prunus mume*) genomes (Velasco et al., 2010; Shulaev et al., 2011; Zhang et al., 2012; Verde et al., 2013; Wu et al., 2013).

The poplar genome was affected by an extra tetraploidization, or whole-genome duplication, which was inferred to have occurred *c.* 60 million of years ago, and which is shared by other Salicaceae plants (Tuskan et al., 2006; Dai et al., 2014). Interestingly, it seems that the poplar genes have mutated at a much slower rate when compared with other eudicots such as Arabidopsis, possibly because poplar, being a tree, has a long generation time (Tuskan et al., 2006; Dai et al., 2014). After polyploidization, the genome often undergoes extensive gene losses and chromosomal rearrangements (Paterson et al., 2004; Wang et al., 2005, 2009). Previous karyotype studies indicate that the modern poplar genome was derived via duplication of the *n* = 12 Salicaceae intermediate, followed by four chromosome fusions and nine chromosome fissions events (Murat et al., 2015). In sum, recursive polyploidizations have produced thousands of duplicated genes, thus providing enormous opportunities for genetic innovation (Taylor and Raes, 2004).

Studies have shown that grape chromosomal rearrangement is relatively less, and compared to poplar and Arabidopsis, it resembles the genome of the common ancestor of the dicotyledonous plants (Jaillon et al., 2007). Therefore, the grape genome is often taken as a reference to understand the genome of other sequenced eudicot plants (Ming et al., 2008; Huang et al., 2009; Schmutz et al., 2010; Velasco et al., 2010; Consortium, 2012). A hierarchical analysis of orthologous and paralogous genes by relating them to temporal events of polyploidizations and speciation can help in understanding the association of polyploidy with structural and functional evolution of the genome (Lyons et al., 2008; Tang et al., 2008b). Multiple comparisons among genomes can clarify their evolution, speciation, and functional innovations (Wang et al., 2015).

**FIGURE 1:**
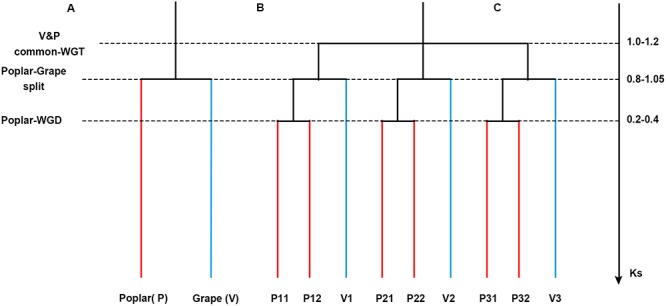
**Phylogenetic tree and gene tree. (A)** A phylogenetic tree. **(B)** A gene tree to show the paralogs in each genome. **(C)** Synonymous substitution values for the events.

To the best of our knowledge, a multiple alignment associated with polyploidy in the poplar genome has not yet been made available. In this study, by taking grape as an outgroup and aligning the genomes of grape and poplar (**Figure 1A**), we were able to build a colinear gene table of homologous genes in each genome and between both, thereby distinguishing orthologous from paralogous gene pairs. Here, based on the alignments of genomes, we then inferred the genomic fractionation involved in the two similar subgenomes of the Salicaceae paleo-tetraploid. We did not include the willow genome in this analysis because its scaffolds are not anchored onto chromosomes; hence, for now, we must leave willow for future study. Nevertheless, the results reported here could provide invaluable genomic material for the community of poplar (and other plant) researchers to investigate evolutionary changes, functional innovations, and phylogenetic structures of gene families and key regulatory pathways.

## Materials and Methods

### Genetic Material

Genomes and their gene annotations for both plant species were downloaded from the Joint Genome Institute (grape genome annotation v.12X, March 2010; poplar genome annotation v.3.0).

### Genomic Homology

By running all-against-all BLASTP, we searched for putative homologous genes (*E*-value < 1e–5; top five matches) within a genome and between genomes. Then, we produced homologous gene dot plots by using a homemade Perl script. In these dot plots, homologous gene pairs were shown in red, blue, and gray to denote the best, second-best, and other matches, respectively, to help distinguish homologies related to different events, recursive polyploidizations, and speciation.

With the information on putative homologous genes as the input, we ran a ColinearScan (Wang et al., 2006) to infer colinear relationships that would reveal homologous blocks within each genome and between genomes. Synonymous nucleotide substitutions per synonymous site (Ks) between colinear genes were estimated by using the Nei-Gojobori approach implemented in the software package, PAML (Yang, 2007). The information on gene collinearity and *Ks* values was also added onto the dot plots.

## Results

### Gene Collinearity within and among Genomes

By using ColinearScan, we could infer intra-genomic homologous genes having collinearity within grape and poplar, respectively, as well as infer the inter-genomic homologs occurring between them. We counted the colinear genes in blocks of apparently different sizes, as measured by colinear gene numbers in blocks (**Table 1**). In grape, 3030 genes were found in 126 blocks that contained more than 10 colinear genes, while in poplar, there were 14 590 colinear genes found in 202 blocks. Considering only the large blocks having more than 50 colinear genes, in grape and poplar there were, respectively, four and 26 blocks involving 384 and 12 521 colinear genes. The largest block in grape had 61 colinear genes located between chromosomes 4 and 18, while the largest block in poplar had 1010 colinear genes located on chromosomes 8 and 10. This result clearly shows that poplar has longer blocks than does grape. Considering now the colinear gene number, the inter-genomic homology between grape and poplar was much better than their intra-genomic homology. We found 561 inter-genomic blocks that had a block size of more than 10 colinear genes each; together, these blocks contained 25 445 colinear genes, of which 14 953 colinear genes came from 103 blocks that had a block size greater than 50 colinear genes (**Table 1**). In sum, we found that there are many more homologs residing on longer blocks between the different genomes than within a genome. A higher similarity between different genomes makes it valuable to perform an inter-genomic comparison to better understand the structure of a genome.

**Table 1:** Number of homologous blocks within a genome or between genomes.

Homologous blocks within and among the genomes		Block length (numbers of collinear genes)					
		>4	>10	>20	>50	LDB^a^	LDBC^b^
Grape	Block	312	126	47	4	61	VV04-VV18
	Genes	4122	3030	2070	384		
Poplar	Block	653	202	86	26	1010	PT08-PT10
	Genes	15879	14590	13723	12521		
Grape vs. Poplar	Block	1723	561	303	103	316	VV18-PT02
	Genes	28934	25445	21902	14953		

^a^*Number of colinear gene pairs occurring in the longest duplicated block (LDB); ^b^LDB on chromosomes (LDBC)*.

### Classification of the Inter-genomic Homology

As noted in the Introduction, after their evolutionary split, poplar underwent an extra whole-genome duplication event whereas grape did not. Therefore, there should be a 1:2 orthologous gene ratio between grape and poplar (**Figure 1**). An orthology was constructed given the grape–poplar split. Specifically, without any gene or DNA loss, we would expect to find a grape gene or chromosomal region having two best matches of orthologous poplar genes or chromosomal regions, and 2 s-best matches of out-paralogous genes or chromosomal regions. An outparalogy was constructed given the whole-genome triplication (WGT) in their eudicot common ancestor. Specifically, as gene or DNA losses tend to occur after polyploidization(s), the expected 1:2 ratio may not hold for all the colinear genes, such that a grape chromosome has two homoeologous chromosomes (or chromosomal regions) due to the WGT, with each having two orthologs that are in effect outparalogs for its homoeologs. In this manner, a grape chromosome would have four out-paralogous chromosomes (or chromosomal regions).

**FIGURE 2:**
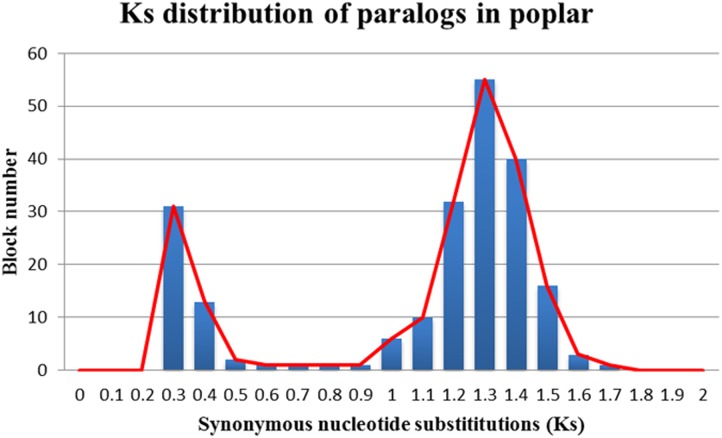
**Ks distribution of paralogs in poplar**. The *x*-axis represents the *Ks*-value range and the *y*-axis represents the number of homologous blocks.

**FIGURE 3:**
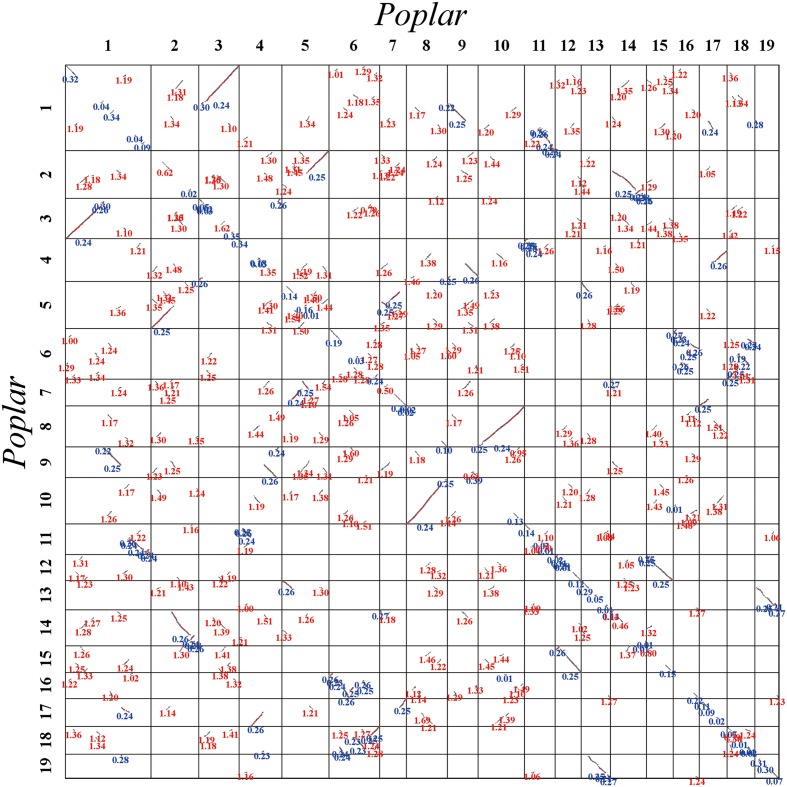
**Dot plot within the poplar genome**. Poplar chromosomes are aligned horizontally and vertically. Homologous gene pairs are shown in red, blue, and gray to denote the best, second-best, and other matches, respectively. The synonymous substitution values (*Ks*) next to the homogeneous block are divided into red and blue according to the *Ks* peak (i.e., duplication event).

**FIGURE 4:**
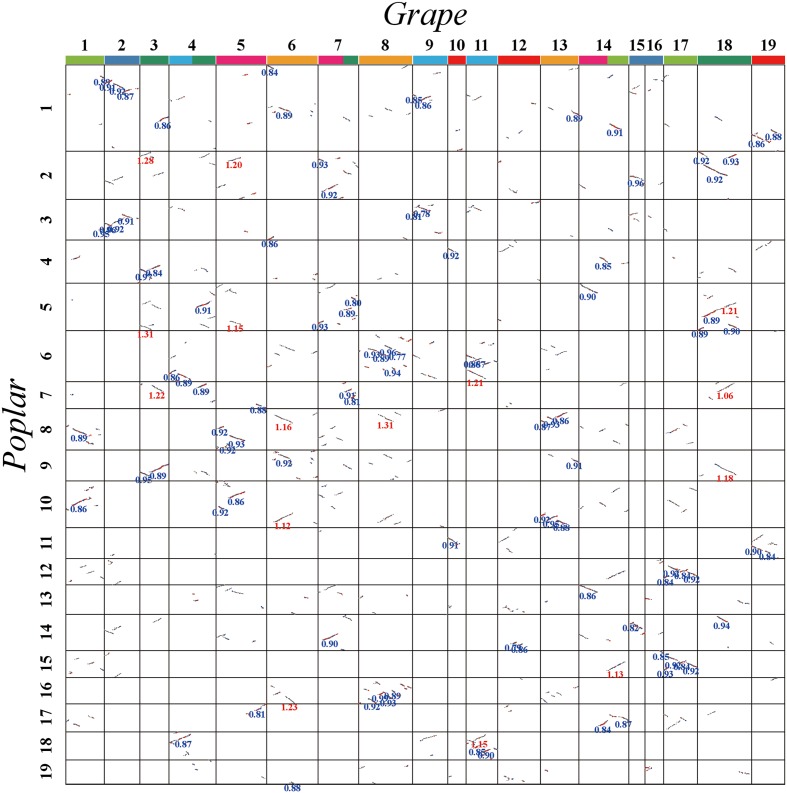
**Dot plot between grape and poplar**. Grape and poplar chromosomes are aligned horizontally and vertically. Grape chromosomes are shown with blocks in seven different colors, to distinguish their origination from seven ancestral chromosomes before the major eudicot common hexaploidy (Jaillon et al., 2007). The same-colored chromosomes, or chromosomal segments, form a triplet of homoeologs produced by the hexaploidy. Homologous gene pairs are shown in red, blue, and gray to denote the best, second-best, and other matches, respectively. The synonymous substitution values (*Ks*) next to the homogeneous block are divided into red and blue according to the obviously separated locations of *Ks* peaks (i.e., divergence event).

The *Ks* values of an intra-genomic homologous block revealed that the poplar-specific whole-genome duplication corresponds to *Ks* ∼0.3+/–0.1, while the eudicot-common WGT event corresponded to 1.3+/–0.3 (**Figure 2**). The *Ks* value of an inter-genomic homologous block further showed that the split of the two plant species corresponds to 0.9+/–0.15. To distinguish those homologous blocks produced by different evolutionary events, the median *Ks* values for the gene pairs in each homologous block were calculated to form dot plots (**Figures 3**, **4**).

**FIGURE 5:**
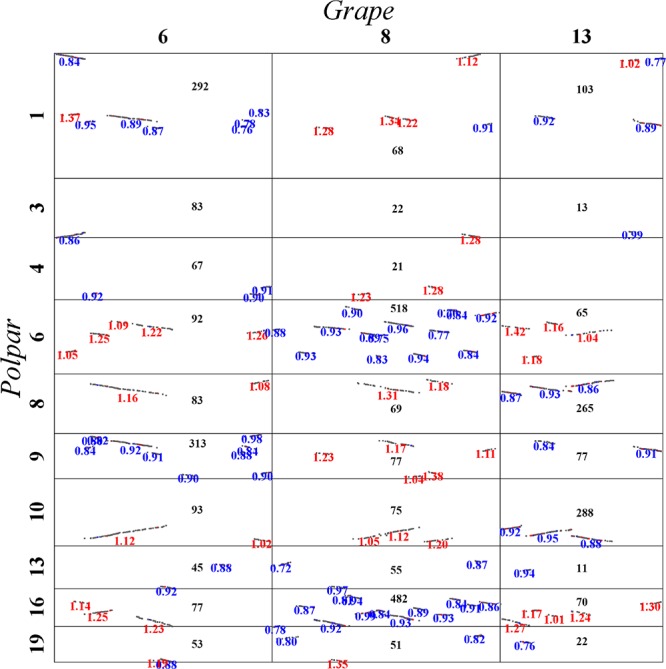
**Local dot plot between grape and poplar**. Grape chromosomes 6, 8, and 13 are homologous chromosomes produced by the hexaploidy common to major eudicot plants.

Here, let us describe in detail how to distinguish the orthologous from the out-paralogous regions between grape and poplar. Grape chromosomes 6, 8, and 13 formed homoeologous triplets in the WGT, and we were able to find their respective two orthologous regions and four out-paralogous regions in poplar (**Figure 5**). This figure displays, between any pair of poplar and grape chromosomes, the accumulated numbers of colinear genes in each homologous block between them. For example, grape chromosome 6 is best matched with, or orthologous to, regions in the poplar chromosomes 1 (292 colinear genes) and 9 (313 colinear genes), each complemented with regions in chromosomes 4 and 3, respectively. Further, grape chromosome 6 has fewer colinear genes with other chromosomes, and those having fewer but an appreciable numbers of colinear genes shared an outparalogy. Comparatively, grape chromosome 8 is best matched with, or orthologous, to regions in the poplar chromosomes 6 (518 colinear genes) and 16 (482 colinear genes); and much of grape chromosome 13 is best matched, or orthologous, to the poplar chromosomes 8 (265 colinear genes) and 10 (288 colinear genes), and its one terminal region is best matched or orthologous to the poplar chromosomes 1 and 9. As mentioned above, the orthologous regions of one homoeolog would be the outparalogs of the other two homoeologs. Besides sharing more colinear genes, these colinear genes on orthologous chromosomes (or regions) have smaller *Ks* values than do the out-paralogous ones. By utilizing a similar strategy, we inferred all the poplar orthologous and out-paralogous regions for each grape chromosome, and we inferred the whole-genome duplication (WGD) paralogs and the WGT paralogs in the poplar genome, and likewise the WGT paralogs in the grape genome.

In sum, we obtained 2423 pairs of WGT paralogs from 86 homoeologous blocks in the grape genome, 6916 WGT paralogs from 292 blocks and 8323 WGD paralogs from 64 blocks in poplar, 11 627 pairs of orthologs from 320 blocks and 6406 pairs of outparalogs from 278 blocks between the poplar and the grape genome. Judging by these above statistics, it seems that poplar has many more WGT paralogs than does grape. This difference likely arose because of the more potential combinations in poplar after an extra WGD, but this does not indicate that poplar has preserved a better genomic structure that resembles the common ancestor of eudicots.

### Multiple Alignments of the Poplar and Grape Genome

With the grape genome as our reference, we produced multiple alignments between the two plant species genomes. A table was set up to store all the inter- and intra-genomic homology information. First, we filled in all grape gene IDs in the first column of the table, then we added the gene IDs from poplar, column by column, according to the inferred gene collinearity. As noted above, in the absence of gene loss, the grape genes would have two colinear orthologous genes in poplar. When the poplar genome contained a gene showing collinearity with a grape gene, a poplar gene ID was put into an appropriate cell in the table. When poplar did not have an expected colinear gene, often due to gene loss or translocation or problematic assembly, a dot (signifying missing) was put into an appropriate cell. Hence, for the two poplar subgenomes, there were 3 (=1 + 2) columns in the table. Additionally, since the core eudicots shared a common WGT (paleo-hexaploidy), each chromosomal segment would be repeated three times. Based on the homology inferred from grape, we therefore extended the table to nine columns. By following this process, we finished constructing a table of colinear genes that reflected both polyploidizations and speciation (**Supplementary Table S1**).

**FIGURE 6:**
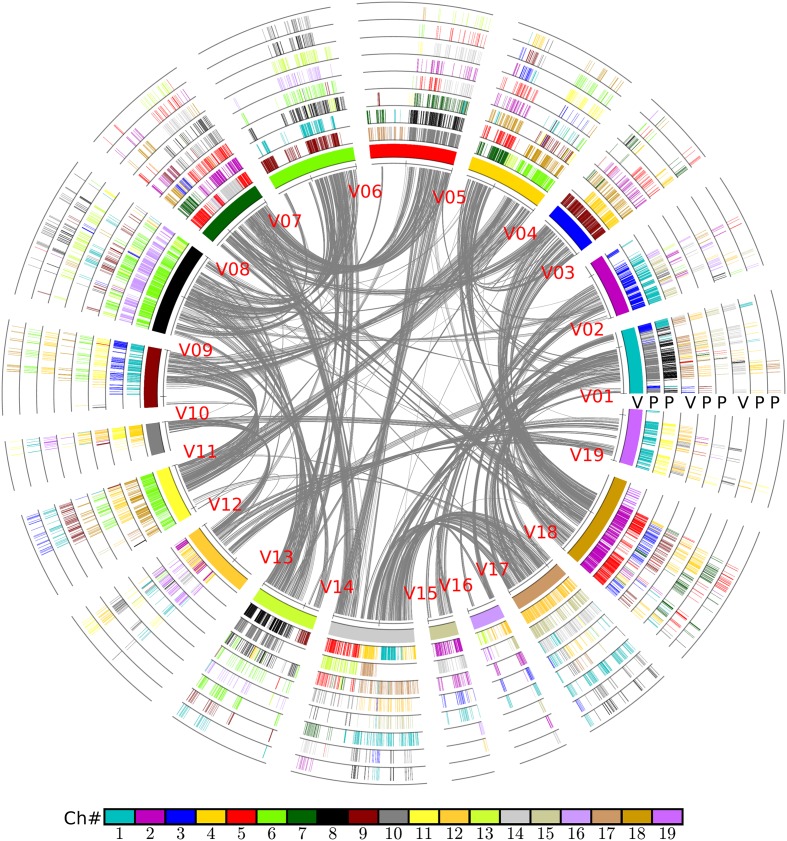
**Alignment of poplar chromosomes with grape as a reference**. Genomic paralogy, orthology, and outparalogy information within and among grape (V) and poplar (P) are displayed in the nine circles; the inner circle represents 19 grape chromosomes, which are differently colored. A grape chromosome block is indicated by short lines, with each representing a gene; a gene short line is colored relative to its source chromosome number in a specific species. A grape genomic region has two sets of poplar-corresponding regions, due to the poplar whole-genome duplication, to form another two circles in sequential order. The shared hexaploidy creates two sets of paralogous regions in grape that form another two circles showing the colinear genes within the grape genome. This second and third grape circle of regions have their own two sets of poplar orthologs that form another two circles of poplar chromosomal regions. The curvy lines in the inner circle show the colinear homologs in grape genome.

**FIGURE 7:**
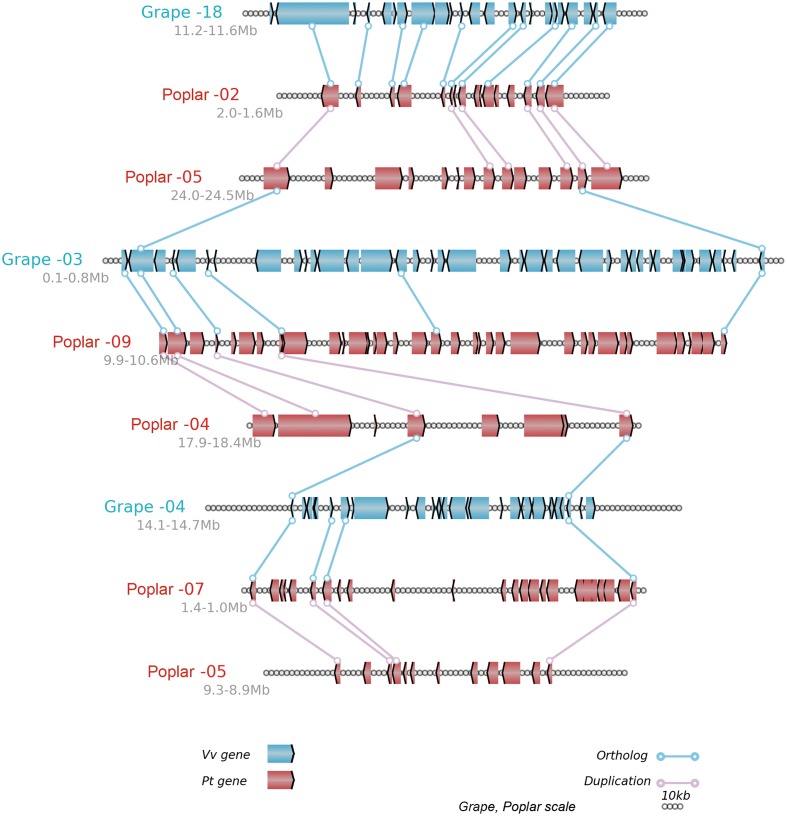
**Alignment of local regions sharing homology**. Vv, Grape; Pt, Poplar; Genes are shown with the pointed boxes showing the transcriptional direction. The homologous genes between neighboring chromosomes (indicated by the straight lines) are linked to lines with circles at their ends.

In brief, the above table summarized the results of the multiple-genome and event-related alignments, thus reflecting layers of tripled and doubled homology due to the recursive polyploidizations, which are displayed in the circles of global multiple alignments (**Figure 6**). Any local region of the global alignment can be linearly displayed to find the details of particular aligned genes (**Figure 7**). For example, as shown in **Figure 7**, the grape homoeologous chromosomes 18, 3, and 4 were related to their respective poplar orthologous regions (and outparalogs). In a short region of 11.2–11.6 Mb on grape chromosome 18, it shares an appreciable orthology with poplar chromosome 2 (1.6–2.0 Mb), which is much better than that seen for poplar chromosome 5 (24.0–24.5 Mb). The corresponding regions of grape chromosome 3 and poplar chromosome 9 contained many more genes. This points to a possible gene deletion, insertion, or other genomic changes in the local regions. A deeper analysis of these local regions is best left to experts in the community.

### Genomic Fractionation in Poplar

Fractionation—the loss of duplicate genes after WGD—causes more gene order disruption than do classical chromosomal rearrangements, and it is particularly prevalent in the flowering plants (Sankoff and Zheng, 2012). Considering the inferred gene collinearity, for 18 837, or 75.65%, of the grape genes, we could not find their colinear orthologs at the expected locations in poplar, with only a tiny fraction of 13.68% having both duplicate copies preserved, and with 10.67% having only one alternative copy preserved in poplar. Conversely, we counted the loss of duplicated genes relative to that of the grape chromosomes. As shown in **Table 2**, for a specific grape chromosome, 58–78% of their genes lack orthologous poplar genes at the expected locations, and 55–73% of their genes lack orthologous poplar genes in both duplicated chromosomal regions. Together, these results indicate that there may be a large number of gene losses in the poplar genome following WGD.

**Table 2:** Poplar gene deletion rate with grape as the reference genome.

Grape		Poplar			
Chromosome	Genes	Paralog 1 lost	Paralog 2 lost	Both Paralogs lost	Loss rate difference
1	1327	0.58	0.63	0.55	0.05
2	1237	0.73	0.7	0.64	0.03
3	1000	0.61	0.63	0.56	0.02
4	1638	0.66	0.71	0.63	0.05
5	1748	0.71	0.68	0.63	0.03
6	1779	0.75	0.62	0.68	0.13
7	1409	0.65	0.7	0.56	0.05
8	1867	0.65	0.69	0.59	0.04
9	1221	0.78	0.78	0.73	0
10	632	0.65	0.7	0.67	0.05
11	1107	0.66	0.74	0.62	0.08
12	1481	0.79	0.71	0.68	0.08
13	1329	0.69	0.67	0.65	0.02
14	1729	0.72	0.73	0.67	0.01
15	561	0.75	0.69	0.68	0.06
16	647	0.58	0.63	0.55	0.05
17	1168	0.73	0.7	0.64	0.03
18	1886	0.61	0.63	0.56	0.02
19	1135	0.66	0.71	0.63	0.05

A notable finding is that the two paralogous regions corresponding to the same grape chromosome often have similar gene loss rates. For 15 of the 19 grape chromosomes, their respective two poplar-duplicated regions have a gene loss rate difference that was ≤0.05, with a pan-genome average of 0.045 (**Table 2**). For example, grape chromosome 9 has nearly the same rates of missing genes in its duplicated poplar orthologous regions, and the largest difference in the rate of missing genes between the poplar-duplicated regions is only 0.13, which involved grape chromosome 6. This result indicated very similar gene loss rates of the two subgenomes inherited from the tetraploid’s progenitor(s), suggesting its likely allotetraploid in nature (as discussed below).

## Discussion

Accumulating evidence supports the view that polyploidizations have contributed to the origination, divergence, and even domestication of land plants (Jiao et al., 2011; Kellogg, 2016). These findings highlight the importance of polyploidizations; however, the biological changes that polyploidizations cause over short or long evolutionary timescales are often elusive to understand. One critical obstacle facing plant scientists is the complex genomes of extant plants, which repack genetic material that was duplicated or triplicated at one time or another into smaller numbers of chromosomes. Irrespective of polyploidization, autopolyploidization, or allopolyploidization, it seems that the genomes of nearly all plants have been subjected to such repacking processes, if we disregard those young polyploids, such as *B. napus* (Chalhoub et al., 2014), tetraploid cottons (Zhang et al., 2015), and bread wheat (Mayer et al., 2014), which may lack enough time to have genetically re-patterned themselves. Nonetheless, quite complicated genome structures have arisen from the recursive repacking processes, which rearrange and merge chromosomes and their segmental regions into fewer chromosomes after a round of polyploidization. Multiple homologous regions co-exist in a genome, and they are divergent in their gene content often after widespread and complement gene losses (Van de Peer, 2004).

To decompose the genome structure—especially the multiple regions of homologous regions in a genome—that has been generated by a certain polyploidization, we must use an outgroup genome that avoided this event. However, a genome that has avoided all known polyploidizations has not yet been found. Instead, we often use grape as a reference genome, since it has preserved well the old genome structure of the eudicot common ancestor before and after a hexaploidization (Jaillon et al., 2007) shared by major eudicots, which includes poplar, the focus of the present study (Jiao et al., 2012). Building on this, the corresponding author and colleagues have developed streamlined software to perform the hierarchical alignment of multiple genomes, which can output a list of genes from different plants to show their orthology or (out) paralogy, as linked to specific speciation(s) or polyploidization(s). These gene lists may prove especially usefully for advancing the evolutionary and phylogenetic analysis of genes and gene families. For example, to date, this streamlined approach was used to carry out a multiple grass genome alignment and evolutionary analysis (Wang et al., 2016a), and to decipher a paleo-decaploid structure of the cotton genome (Wang et al., 2016b).

In the present study, with grape as a reference, we aligned the genome of poplar to that of grape to produce a gene table of paralogs and orthologs, which should benefit those in the Salicaceae research community and beyond. Notably, we found that poplar has two very similar subgenomes in terms of gene loss (retention) rates that resulted from the Salicaceae-common tetraploidization (Tuskan et al., 2006). The high similarity in gene loss (and retention) rates involved the majority of, if not all, chromosomes. This result is in stark contrast to maize: it was a paleo-tetraploid plant ∼26 million years ago (Wang et al., 2016a), and its two extant subgenomes have significantly divergent gene loss (retention) rates amounting to a ∼20% difference (Schnable et al., 2011). Coupled to the evidence of gene expression, the observation of unbalanced gene losses in maize was explained by a dominant subgenome merging with a sensitive one, and the ensuing event was allotetraploidization. However, the Salicaceae-common tetraploidization occurred ∼60 million years ago (Tuskan et al., 2006) and is much older than that seen in maize. Yet, since then, and in twice the elapsed time available to maize, the poplar subgenomes have been well preserved their gene contents and ancestral genomic structure, and share a pan-subgenome high similarity. That is, any subgenome found in poplar did not dominate over any other. Hence, this result raises the intriguing possibility of autopolyploidization in the Salicaceae common ancestor. This hypothesis could well explain our present results, in that the tetralets of chromosomes might have recombined with one another through multivalent pairing, thereby maintaining their similarity over a long period of evolutionary time. Then a diploidization process occurred to recover the diploid hybridity, allowing for homologous regions to diverge (Wang et al., 2005).

## Author Contributions

XW conceived the study and led the research. JW implemented and coordinated the analysis. YLiu, WG, ZW, YLi, NY, SS, and LZ performed the analysis. WG and JW contributed the tools for analysis. JW, WG, NY, and SS performed the analysis and provided constructive discussions. XW, JW, and YLiu wrote the paper.

## Conflict of Interest Statement

The authors declare that the research was conducted in the absence of any commercial or financial relationships that could be construed as a potential conflict of interest.

## Supplementary Material

TABLE S1 **Homologous alignment of grape and poplar genomes**.
